# Supplementary material for: The Development of a Regulator of Human Serine Racemase for N-Methyl-D-aspartate Function
Source: Biomedicines. 2024 Apr 12;12(4):853. doi: 10.3390/biomedicines12040853 (PMC11048566; doi:10.3390/biomedicines12040853)
Supplement: Supplementary file 1 [file biomedicines-12-00853-s001.zip › biomedicines-2928341-supplementary.pdf]

Supporting information content:

Figure S1: Recombinant wild type hSR

Scheme S1: Synthesis form 3,4,5-trihydroxybenzoic acid to 7-(allyloxy)-2,2-diphenylbenzo[d][1,3]dioxole-5-carbonyl chloride._

Scheme S2: Synthesis from 7-(allyloxy)-2,2-diphenylbenzo[d][1,3]dioxole-5-carbonyl chloride to 7-((7-(Benzyloxy)-2,2-diphenylbenzo[d][1,3]dioxole-5-carbonyl)oxy)-2,2-diphenylbenzo[d][1,3]dioxole-5-carboxylic acid.

Scheme S3: Synthesis from 7-(allyloxy)-2,2-diphenylbenzo[d][1,3]dioxole-5-carbonyl chloride to (2*R*,3*R*,4*S*,5*R*)-tetrahydro-2*H*-pyran-2,3,4,5-tetrayl tetrakis(3-((3,4-dihydroxy-5-((3,4,5-trihydroxybenzoyl)oxy)benzoyl)ox)-4,5-dihydroxybenzoate) (α12G).

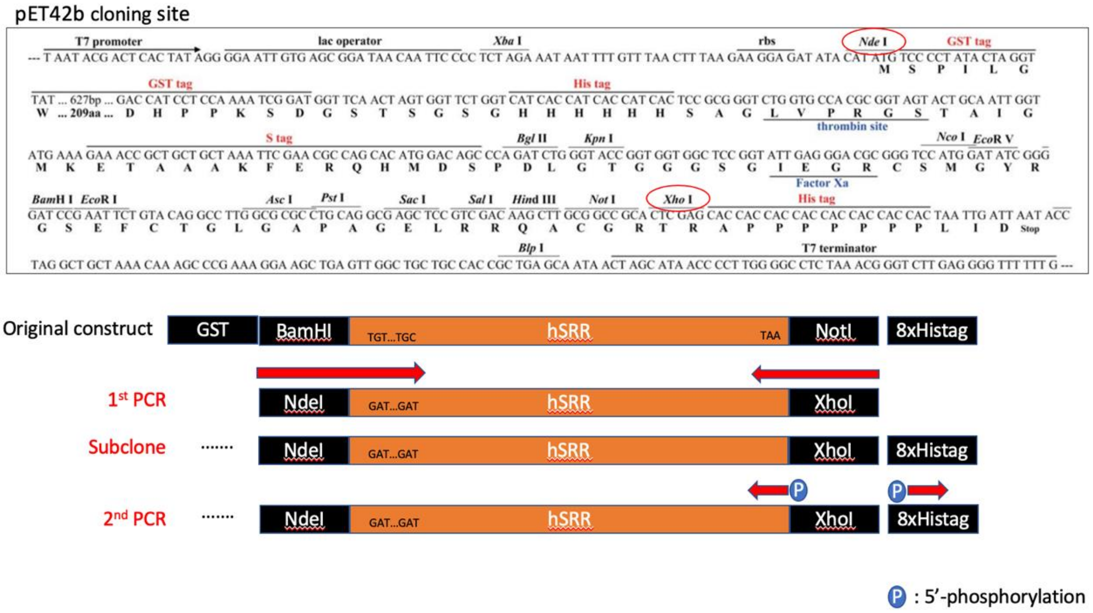

Figure S1

Recombinant wild-type hSR was constructed with a 6-histidine tag at the 3' terminus. In detail, the original construct was subcloned into pET42b using the 5'BamHI and 3'NotI restriction sites. The first PCR amplified hSR by introducing 5'NdeI and 3'XhoI sites and skipping the stop codon. The PCR amplicon was then subcloned into pET42b cleaved by NdeI and XhoI restriction sites, which removed all tags at the 5' terminus of pET42b. The second PCR step amplified the whole plasmid using a set of 5'phosphorylation primers that skipped 6 nucleotides of the XhoI site and 2 histidine tags out of 8 histidine tags of pET42b to generate the correct sequence.

General method

All moisture and oxygen sensitive reactions were conducted in oven-dried glassware under N_2 protection. Acetonitrile, dichloromethane (DCM), methanol, methyl ethyl ketone, anhydrous tetrahydrofuran (THF), pyridine, and ethyl acetate (EtOAc) were used as received. Commercially available agents were purchased and used as received, unless otherwise noted. Column chromatography was carried out under positive pressure using spherical silica gel 40/75 µm (Fuji). The reactions were monitored by thin layer chromatography (TLC), which was performed on precoated silica gel 60 F-254 plates (Merck). Spots were visualized by exposure to UV light. ^{1}H-NMR spectra of samples in the indicated solvent were recorded on 400 or 500 MHz on Bruker Avance 400 or NEO 500 spectrometer, respectively. Chemical shifts are given in ppm (δ) comparatively to the residual solvent signal, which was used as an internal reference. Coupling constants (J) are given in Hertz (Hz), and the following abbreviations are used

to describe the signal multiplicity: s (singlet), bs (broad singlet), d (doublet), t (triplet), q (quadruplet), m (multiplet).

Synthesis of α12G

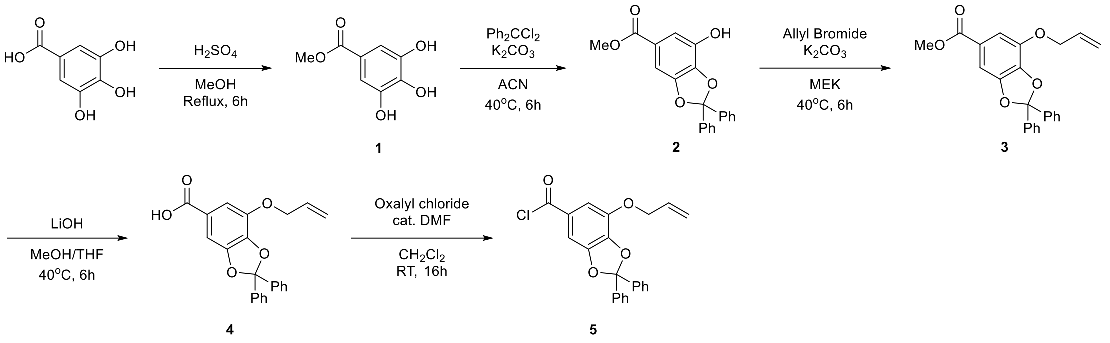

Scheme S1.

Methyl 3,4,5-trihydroxybenzoate (1)

To a solution of 3,4,5-trihydroxybenzoic acid (10.0 g, 58.8 mmol) in methanol (118.0 mL) at RT was added sulfuric acid (3.1 mL, 58.8 mmol). The resulting mixture was heated to reflux for 6h. After the reaction was complete, the reaction mixture was concentrated under vacuum. The residue was diluted with EtOAc and partitioned with water. The organic layer was washed with brine, dried over anhydrous magnesium sulfate, and filtered. The filtrate was concentrated in vacuo to afford methyl 3,4,5-trihydroxybenzoate as a white solid (9.6 g, 89%). ^{1}H NMR (CD_3OD, 400MHz) δ 7.03 (s, 2H), 3.81 (s, 3H).

Methyl 7-hydroxy-2,2-diphenylbenzo[d][1,3]dioxole-5-carboxylate (2)

To a solution of 3,4,5-trihydroxybenzoate (**1**) (10.0 g, 54.3 mol) in acetonitrile (543.0 mL) was added potassium carbonate (15.0 g, 108.6 mmol) and α,α-dichlorodiphenylmethane (9.9 mL, 51.6 mmol). The mixture was stirred at 40°C for 6h. After the reaction was complete, the mixture was concentrated under vacuum. The residue was diluted with dichloromethane and partitioned with water. The organic layer was washed with brine, dried over anhydrous magnesium sulfate, and filtered. The filtrate was concentrated in vacuo. The resulting residue was purified by flash column chromatography on silica gel with EtOAc/hexanes (1:3) to afford **2** as a white solid (10.5 g, 55%). ^{1}H NMR ($CDCl_3$, 400 MHz) δ 7.57-7.55 (m, 4H), 7.39-7.34 (m, 7H), 7.20 (s, 1H), 3.84 (s, 3H).

Methyl 7-(allyloxy)-2,2-diphenylbenzo[d][1,3]dioxole-5-carboxylate (3)

To a solution of compound **2** (10.0 g, 28.7 mmol) in methyl ethyl ketone (144.0 mL)

was added potassium carbonate (7.9 g, 57.4 mmol) and allyl bromide (8.7 mL, 100.5 mmol). The mixture was stirred at 40°C for 6h. After the reaction was complete, the mixture was concentrated in vacuo. The residue was diluted with dichloromethane, and partitioned with water. The organic layer was washed with brine, dried over anhydrous magnesium sulfate, and filtered. The filtrate was stripped down in vacuo. The residue was purified by flash column chromatography on silica gel with EtOAc/hexanes (1:4) to afford **3** as a white solid (10.4 g, 93%). ^1H NMR ($CDCl_3$, 400 MHz) δ 7.59-7.57 (m, 4H), 7.37 (d, J = 5.2 Hz, 6H), 7.32 (s, 1H), 7.26 (s, 1H), 6.09-6.02 (m, 1H), 5.40 (d, J = 17.2 Hz, 1H), 5.28 (d, J = 10.5 Hz, 1H), 4.70 (d, J = 5.4 Hz, 2H), 3.85 (s, 3H).

7-(Allyloxy)-2,2-diphenylbenzo[d][1,3]dioxole-5-carboxylic acid (4)

To a solution of compound **3** (10.0 g, 28.7 mmol) in methanol/tetrahydrofuran (1:1, 102.0 mL) was added lithium hydroxide (1.2 g, 51.5 mmol). The resulting mixture was stirred at 40°C for 6h. The mixture was concentrated under vacuum. The resulting residue was made acidic (pH = 5) with the dropwise addition of 10% hydrochloric acid. The solid was collected and purified by recrystallization with EtOAc/hexanes (1:4) to **4** as a white solid (9.0 g, 93%). ^1H NMR ($CDCl_3$, 400 MHz) δ 7.60-7.58 (m, 4H), 7.38-7.37 (m, 7H), 7.32 (s, 1H), 6.11-6.01 (m, 1H), 5.41 (d, J = 17.2 Hz, 1H), 5.29 (d, J = 10.8 Hz, 1H), 4.71 (d, J = 5.2 Hz, 2H).

7-(Allyloxy)-2,2-diphenylbenzo[d][1,3]dioxole-5-carbonyl chloride (5)

To a stirring solution of compound **4** (9.0 g, 24.0 mmol) in dichloromethane (120.0 mL) was added oxalyl chloride (6.2 mL, 72.1 mmol) and DMF (0.1 mL) at 0°C. The mixture was stirred at RT for 16h. The mixture was concentrated under vacuum to afford **5** (9.1g, crude) as a yellow solid. ^1H NMR ($CDCl_3$, 400 MHz) δ 7.59-7.58 (m, 4H), 7.42-7.39 (m, 8H), 6.11-6.01 (m, 1H), 5.44 (dd, J = 17.2, 1.2 Hz, 1H), 5.33 (dd, J = 10.4, 0.9 Hz, 1H), 4.73 (d, J = 5.4 Hz, 2H).

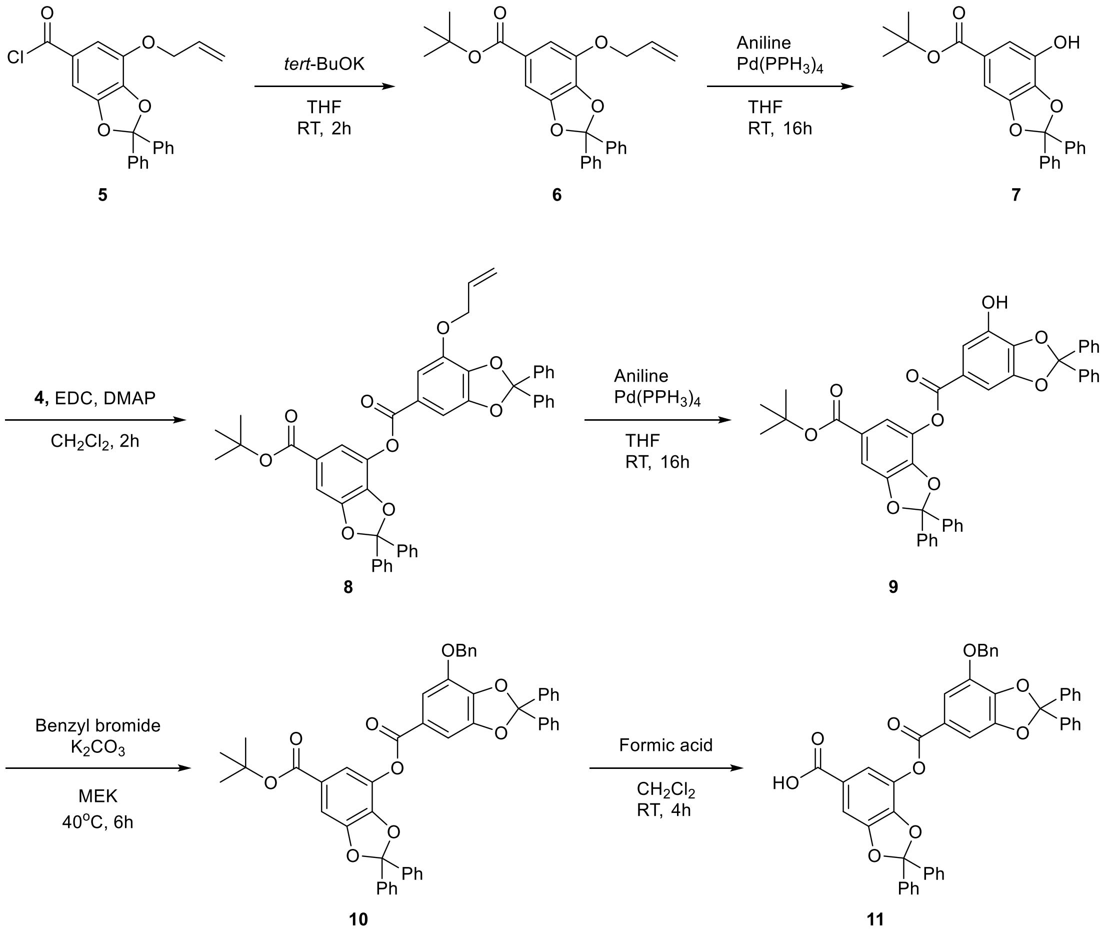

Scheme S2.

tert-Butyl 7-(allyloxy)-2,2-diphenylbenzo[d][1,3]dioxole-5-carboxylate (6)

To a solution of compound **5** (30.0 g, 76.5 mmol) in tetrahydrofuran (300.0 mL) was added potassium *tert*-butoxide (10.3 g, 91.8 mmol) solution in tetrahydrofuran (100 mL) under N_2 at 0°C. The mixture was stirred at RT for 2h. After the reaction was complete, the residue was diluted with EtOAc/water. The organic layer was washed with brine, dried over anhydrous magnesium sulfate, and filtered. The filtrate was concentrated under vacuum. The residue was purified by flash column chromatography on silica gel with EtOAc/hexanes (1: 8) to afford **6** as a white solid (32 g, 97%). ^{1}H NMR ($CDCl_3$, 400 MHz) δ 7.60-7.57 (m, 4H), 7.39-7.35 (m, 6H), 7.29-7.28 (d, J = 1.2 Hz, 1H), 7.22-7.21 (d, J = 1.2 Hz, 1H), 6.11-6.01 (m, 1H), 5.42-5.38 (dd, J = 17.2, 1.5 Hz, 1H), 5.29-5.26 (dd, J = 10.5, 1.3 Hz, 1H), 4.71-4.69 (d, J = 5.6 Hz, 2H), 1.55 (s, 9H).

tert-Butyl 7-hydroxy-2,2-diphenylbenzo[d][1,3]dioxole-5-carboxylate (7)

To a stirred solution of compound **6** (32.0 g, 74.3 mmol) in anhydrous tetrahydrofuran (766.0 mL) was added aniline (5.2 mL, 37.2 mmol) and

tetrakis(triphenylphosphine)palladium (8.6 g, 7.43 mmol). The mixture was stirred at RT under N_2 for 16h. The mixture was filtered through a bed of Celite and the filtrate was concentrated in vacuo. The residue was purified by flash column chromatography on silica gel with EtOAc/hexanes (1: 4) to afford **7** (27 g, 93%) as a white solid. ^{1}H NMR ($CDCl_3$, 400 MHz) δ 7.61-7.58 (m, 4H), 7.40-7.38 (m, 7H), 7.19-7.18 (d, J = 1.4 Hz, 1H), 6.14 (br, 1H), 1.58 (s, 9H).

6-(*tert*-Butoxycarbonyl)-2,2-diphenylbenzo[d][1,3]dioxol-4-yl-7-(allyloxy)-2, 2-diphenylbenzo[d][1,3]dioxole-5-carboxylate (8)

A mixture of the compound **7** (27 g, 69.2 mmol), compound **4** (27.2 g, 72.6 mmol) and 4-dimethylaminopyridine (0.84 g, 6.9 mmol) in dichloromethane (692.0 mL) was stirred at 0°C and added with 1-ethyl-3-(3-dimethyl-aminopropyl)carbodiimide (14.6g, 76.1 mmol). The mixture was stirred 10 mins at 0°C then stirred at RT. After the reaction was complete, the mixture was extracted with DCM/water, washed with brine, dried over anhydrous magnesium sulfate, and filtered. The filtrate was evaporated in vacuo. The residue was purified by flash column chromatography on silica gel with EtOAc/hexanes (1: 9) to afford the compound **8** (48.2 g, 93%) as a white solid. ^{1}H NMR ($CDCl_3$, 400 MHz) δ 7.64-7.61 (m, 4H) , 7.59-7.56 (m, 4H) , 7.54-7.53 (d, J = 1.5 Hz, 1H) , 7.50-7.49 (d, J = 1.5 Hz, 1H) , 7.47-7.45 (m, 2H) , 7.43-7.38 (m, 12H) , 6.16-6.06 (m, 1H) , 5.48-5.43 (dd, J = 17.2, 1.4 Hz, 1H) , 5.34-5.31 (dd, J = 10.4, 1.2 Hz, 1H) , 4.78-4.76 (d, J = 5.5 Hz, 2H) , 1.57 (s, 9H) .

6-(*tert*-Butoxycarbonyl)-2,2-diphenylbenzo[d][1,3]dioxol-4-yl-7-hydroxy-2,2-diphenylbenzo[d][1,3]dioxole-5-carboxylate (9)

To a stirred solution of compound **8** (20.0 g, 26.8 mmol) in anhydrous tetrahydrofuran (267.8 mL) was added aniline (1.9 mL, 13.4 mmol) and tetrakis(triphenylphosphine)palladium (3.1 g, 2.7 mmol). The mixture was stirred at RT under N_2 protection for 16h. The mixture was filtered through a bed of Celite and the filtrate was concentrated in vacuo. The residue was purified by flash column chromatography on silica gel with EtOAc/hexanes (1:5) to afford the **9** as a white solid (17.5 g, 92%). ^{1}H NMR ($CDCl_3$, 400 MHz) δ 7.62-7.58 (m, 4H),7.58-7.54 (m, 4H), 7.52-7.51 (d, J = 1.4 Hz, 1H), 7.46 (s, 1H), 7.45 (s, 1H), 7.43-7.36 (m, 13H), 5.67 (br, 1H), 1.56(s, 9H).

6-(*tert*-Butorycarbonyl)-2,2-diphenytbenzo[d][1,3]dioxol-4-yl-7-(benzylary)-2,2-diphenylbenzo[d][1,3]dioxole-5-carboxylate (10)

To a solution of compound **9** (2.5 g, 3.5 mmol) in methyl ethyl ketone (35.4 mL) was added potassium carbonate (1.5 g, 10.6 mmol) and benzyl bromide (1.3 mL, 10.6

mmol). The mixture was stirred at 40°C for 6h. After the reaction was complete, the mixture was concentrated in vacuo. The residue was diluted with dichloromethane and partitioned with water. The organic layer was washed with brine, dried over anhydrous magnesium sulfate, and filtered. The filtrate was stripped down in vacuo. The residue was purified by flash column chromatography on silica gel with EtOAc/hexanes (1:8) to afford the **10** as a white solid (2.6 g, 92%). ^{1}H NMR ($CDCl_3$, 400 MHz) *δ* 7.61-7.55 (m, 9H), 7.48-7.33 (m, 20H), 5.29 (s, 2H), 1.56 (s, 9H).

7-((7-(Benzyloxy)-2,2-diphenylbenzo[d][1,3]dioxole-5-carbonyl)oxy)-2,2-diphenylbenzo[d][1,3]dioxole-5-carboxylic acid (11)

To a stirred solution of the compound **10** (2.5 g, 3.1 mmol) in anhydrous dichloromethane (31.4 mL) was added formic acid (31.4 mL) at 0°C. After stirring at 0°C for 10 mins, the reaction mixture was stirred under RT for 4h. The mixture was washed with water 3 times and brine, dried over anhydrous magnesium sulfate, and filtered. The filtrate was evaporated in vacuo. The residue was purified by flash column chromatography on silica gel with EtOAc/dichloromethane (10%) to afford the **11** (1.3 g, 60%) as a white solid. ^{1}H NMR ($CDCl_3$, 400 MHz) *δ* 7.60-7.53 (m, 10H), 7.52-7.51 (d, *J* = 1.5 Hz, 1H), 7.47-7.45 (m, 3H), 7.41-7.32 (m, 15H), 5.28 (s, 2H).

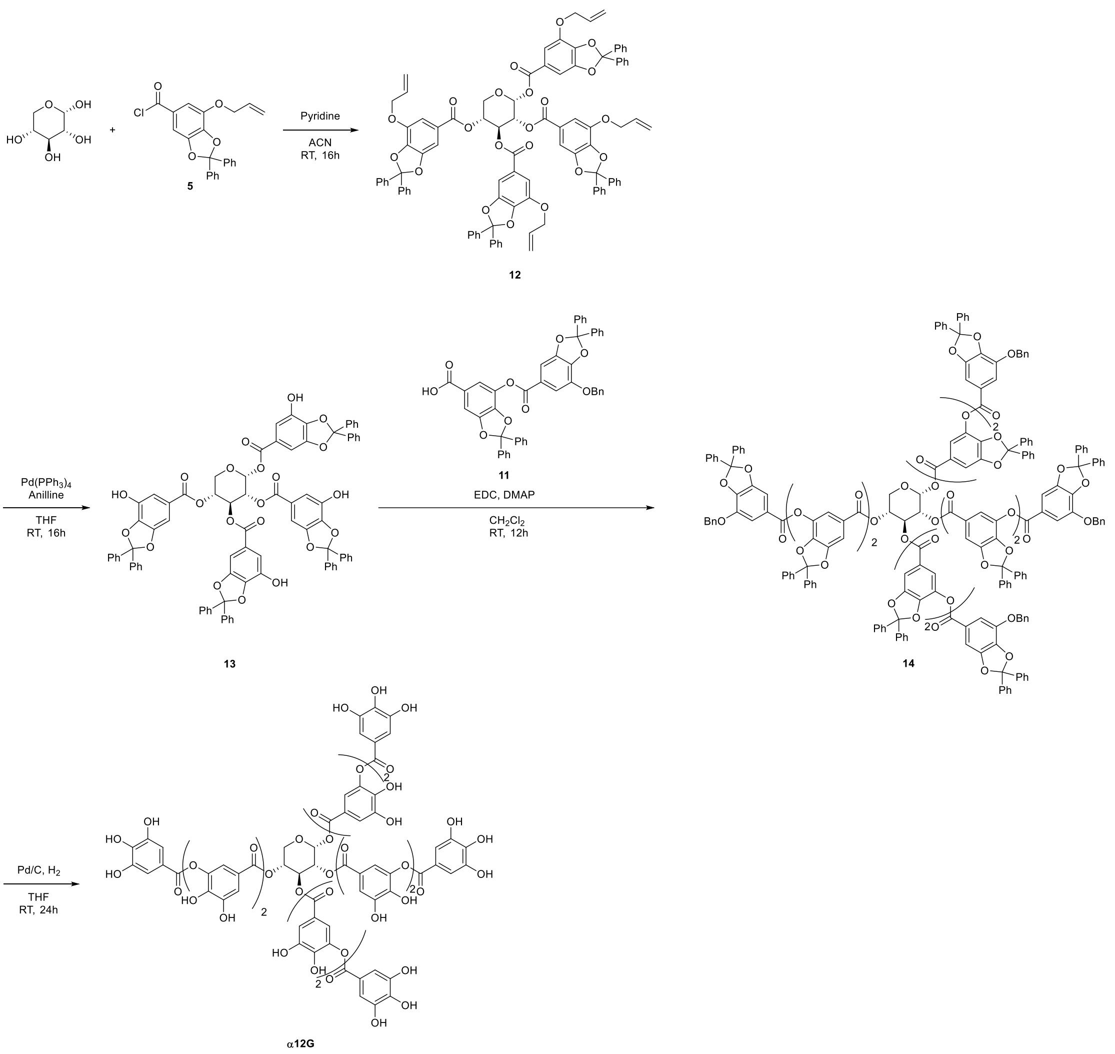

Scheme S3.

(2*R*,3*R*,4*S*,5*R*)-tetrahydro-2*H*-pyran-2,3,4,5-tetrayl tetrakis(7-(allyloxy)-2,2-diphenylbenzo[d][1,3]dioxole-5-carboxylate) (12)

To a slurry solution of the α-D-(+)-xylose (1.0 g, 6.7 mmol), compound **5** (11.8 g, 30.0 mmol) in anhydrous acetonitrile (33.0 mL) was added anhydrous pyridine (4.7 mL, 60.0 mmol) at 0°C. The reaction mixture was stirred under RT for 16h. The crude mixture was cooled to 0°C, quenched with 1N hydrochloric acid, and extracted with EtOAc. The slurry organic layer was washed with brine, filtered, and separated. The organic layer was dried over anhydrous magnesium sulfate and filtered. The organic solution was evaporated in vacuum purified by flash column chromatography on silica gel with DCM/hexanes (50%~70%) to afford the **12** (8.9 g, 85%) as a white bubble form solid. ^{1}H NMR ($CDCl_3$, 500 MHz) δ 7.65-7.58 (m, 4H), 7.58-7.46 (m, 12H), 7.41-7.28 (m, 27H), 7.23-7.20 (m, 2H), 7.18- 7.13 (m, 2H), 7.11 (pseudo d, *J* = 1.4 Hz, 1H), 6.63 (d, *J* = 3.7 Hz, 1H), 6.12-5.92 (m, 4H), 5.90-5.78 (m, 1H), 5.47-5.17 (m, 9H), 5.13-5.07 (m, 1H), 4.76-4.70 (m, 2H), 4.69-4.57 (m, 4 HD) , 4.48-4.42 (m ,2H) , 4.21 (dd, *J* =11.2, 5.8 Hz,

1H) , 3.90 (t, *J* =11.0 Hz, 1H).

(2*R*,3*R*,4*S*,5*R*)-tetrahydro-2*H*-pyran-2,3,4,5-tetrayl tetrakis(7-hydroxy-2,2-diphenylbenzod][1,3]dioxole-5-carboxylate) (13)

To an argon flushed solution of the compound **12** (8.9 g, 5.65 mmol) and tetrakis(triphenylphosphine)palladium (659 mg, 0.56 mmol) in dry tetrahydrofuran (113 mL), aniline (1.56 mL, 16.95 mmol) was added and stirred under RT 16h. The mixture was extracted with DCM and partitioned with 1 N hydrochloric acid. The organic layer was washed with brine, dried over magnesium sulfate, and filtered. The organic solvent was evaporated and purified by flash column chromatography on silica gel with EtOAc/DCM = 0%~ 10%. The residue was precipitated with DCM/hexanes ~10% to afford the **13** as an off-white solid (7.4 g, 92%). ^{1}H NMR ($CDCl_3$, 400 MHz) δ 7.61-7.41 (m, 17H), 7.40-7.26 (m, 23H), 7.25-7.03 (m, 8H), 6.54 (d, *J* = 3.5 Hz, 1H), 6.05 (t, *J* = 9.9 Hz, 1H), 5.42 (dd, *J* = 10.2, 3.5 Hz, 1H), 5.39-5.32 (m, 1H), 4.19-4.07 (m, 1H), 3.85 (t, *J* = 11.0 Hz, 1H).

(2*R*,3*R*,4*S*,5*R*)-tetrahydro-2*H*-pyran-2,3,4,5-tetrayl tetrakis(7-((7-((7-(benzyloxy)-2,2-diphenylbenzo[d][1,3]dioxole-5-carbonyl)oxy)-2,2-diphenylbenzo[d][1,3]dioxole-5-carbonyl)oxy)-2,2-diphenylbenzo[d][1,3]dioxole-5-carboxylate) (14)

To a slurry solution of the compound **13** (200 mg, 0.14 mmol), compound **11** (440 mg, 0.59 mmol) and 4-dimethylaminopyridine (10 mg, 0.08 mmol) in DCM (6.4 mL) at 0°C, 1-ethyl-3-(3-dimethylaminopropyl)carbodiimide (126 mg, 0.65 mmol) was added and stirred at RT for 12h. The crude mixture was extracted with DCM/water and washed with brine. The organic layer was dried over anhydrous magnesium sulfate, filtered, and evaporated in vacuo. The residue was purified by flash column chromatography on silica gel with DCM/hexanes = 60%~80% and precipitate with DCM/hexanes ~ 10% to afford **14** (500 mg, 82%) as an off-white solid. ^{1}H NMR ($CDCl_3$, 400 MHz) δ 7.71-7. 12 (m, 164H), 6.65 (d, *J* = 3.7 Hz, 1H), 6.07 (t, *J* = 9.9 Hz, 1H), 5.44 (dd, *J* = 10.3, 4.0 Hz, 1H), 5.42-5.33 (m, 1H), 5.24-5.12 (m, 6H), 5.06 (s, 2H), 4.15 (dd, *J* = 11.0, 5.3 Hz, 1H), 3.90 (t, *J* = 11.0 Hz, 1H).

(2*R*,3*R*,4*S*,5*R*)-tetrahydro-2*H*-pyran-2,3,4,5-tetrayl tetrakis(3-((3,4-dihydroxy-5-((3,4,5-trihydroxybenzoyl)oxy)benzoyl)ox)-4,5-dihydroxybenzoate) (α12G)

To a solution of the compound **14** (505 mg, 0.12 mmol) in anhydrous tetrahydrofuran (10.0 mL), the dried 10 wt% Pd/C solid (100 mg) was added. The mixture was stirred at RT under H_2 (8 atm) for 24h. The crude mixture was filtered, washed with tetrahydrofuran and EtOAc, and the combined filtrates were evaporated in vacuo. The residue was re-dissolved in EtOAc. The organic layer was washed by 1N hydrochloric

acid and brine. The organic residue was dried over magnesium sulfate, filtered, and evaporated in vacuum. The residue was precipitated with EtOAc/hexanes (10%). The solid crude was further purified by reverse phase C_{18} flash column with ACN/H_20 (25%-40% with 1% formic acid as additive). The collected residue was extracted with EtOAc, and the organic layer was washed with brine. The organic layer was dried over magnesium sulfate, filtered, evaporated, and precipitated with EtOAc/n-pentane (~ 10%) to afford α12G as an off-white solid (145 mg, 63%). ^1H NMR (CD_3OD, 400 MHz) δ 7.65-7.15 (m, 22H), 7.15-6.94 (m, 2H), 6.76-6.62 (m, 1H), 6.19-6.07 (m, 1H), 5.65-5.45 (m, 2H), 4.33-4.17 (m, 1H), 4.17-3.97 (m, 1H).
